# genCRC32: collision-free CRC32-based hashing of DNA sequences

**DOI:** 10.1093/bioadv/vbaf315

**Published:** 2025-12-04

**Authors:** Pavel Beran, Michael Rost, Kristina Beranová, Martin Kváč, Dagmar Stehlíková, Obiora Emmanuel Udoh, Eva Jozová, Vladislav Čurn

**Affiliations:** Faculty of Agriculture and Technology, University of South Bohemia in České Budějovice, České Budějovice 370 05, Czech Republic; Faculty of Agriculture and Technology, University of South Bohemia in České Budějovice, České Budějovice 370 05, Czech Republic; Faculty of Agriculture and Technology, University of South Bohemia in České Budějovice, České Budějovice 370 05, Czech Republic; Faculty of Agriculture and Technology, University of South Bohemia in České Budějovice, České Budějovice 370 05, Czech Republic; Institute of Parasitology, Biology Centre of the Academy of Sciences of the Czech Republic, České Budějovice 370 05, Czech Republic; Faculty of Agriculture and Technology, University of South Bohemia in České Budějovice, České Budějovice 370 05, Czech Republic; Faculty of Agriculture and Technology, University of South Bohemia in České Budějovice, České Budějovice 370 05, Czech Republic; Faculty of Agriculture and Technology, University of South Bohemia in České Budějovice, České Budějovice 370 05, Czech Republic; Faculty of Agriculture and Technology, University of South Bohemia in České Budějovice, České Budějovice 370 05, Czech Republic

## Abstract

**Motivation:**

Efficient and collision-free hashing of DNA sequences is essential for accuracy and performance in bioinformatics applications such as genome assembly, sequence alignment, and metagenomic classification. Traditional hashing methods often result in collisions, impacting the precision and/or performance of downstream analyses. Thus, it is highly advantageous to have hashing functions that guarantee collision-free mappings for DNA sequences, particularly for k-mers up to length 16, where practical limits for 32-bit hashing are reached. In this study, we evaluate genCRC32 as a hashing primitive, reporting collision behavior, bucket balance, sensitivity to single-base changes, and speed to inform its potential use in downstream tools. Evaluation within specific software tools is outside the scope of this paper and is planned as future work.

**Results:**

We present genCRC32, an innovative hashing method that integrates a straightforward preprocessing step (gen32) with CRC32 hashing, specifically identifying eight CRC32 polynomials that ensure collision-free hashing for all DNA k-mers up to 16 nucleotides in length. Through extensive empirical evaluations, genCRC32 demonstrated zero collisions for these k-mers, achieving a one-to-one mapping without auxiliary data structures. Benchmark tests confirmed minimal computational overhead introduced by preprocessing, maintaining hashing performance comparable to established methods such as MurmurHash3 and xxHash32.

**Availability and implementation:**

The source code for genCRC32 is publicly available at: https://github.com/berybox/genCRC32. The implementation is provided in Go (version 1.24) and leverages only standard libraries, ensuring portability and ease of integration into existing bioinformatics workflows.

## 1 Introduction

Efficient management and rapid querying of large-scale genomic data are fundamental challenges in contemporary bioinformatics. Hash functions are algorithms that map variable-length inputs to fixed-size outputs. They are extensively employed to accelerate key tasks such as sequence alignment, genome assembly, and database searches by enabling constant-time lookups and compact data representations (Rivest 1992, Menezes *et al.* 1996, Ramakrishna and Zobel 1997, Marchet *et al.* 2021, Pibiri 2022). However, conventional hash functions inherently permit collisions—instances where distinct inputs yield identical hash values—with potentially detrimental effects on accuracy and performance in applications requiring unambiguous sequence identification (Dietzfelbinger *et al.* 1994, Majewski *et al.* 1996).

DNA k-mer hashing is particularly sensitive to collisions. Although a 32-bit hash space can theoretically encode up to 416 (≈ 232) distinct 16-mers without collision, in practice collisions occur far more frequently due to uneven mapping and distribution properties of typical hash functions. These collisions can lead to misassemblies in de Bruijn graph construction, erroneous variant calls, and decreased sensitivity in metagenomic classification (Altschul *et al.* 1990, Phillippy *et al.* 2008). To address collisions, one might consider minimum perfect hash functions. Minimum perfect hash functions (MPHFs) guarantee collision-free O(1) lookups on static key sets, and modern constructions are compact and scalable—combining near-optimal space with fast queries and practical builds (Esposito *et al.* 2020, Pibiri and Trani 2021, Lehmann *et al.* 2025, Beling and Sanders 2025). However, being set-specific, they are less suited to our goal of a general-purpose 32-bit hash, which motivates the CRC32-based approach.

Cyclic redundancy check (CRC) codes—ubiquitous in digital communications for error detection—offer an attractive balance of simplicity, hardware acceleration, and strong bit‐diffusion properties (Peterson and Brown 1961, Koopman and Chakravarty 2004). In particular, CRC32 applies a 32-bit polynomial divisor to produce checksums that detect common data corruptions with minimal computational overhead (Castagnoli *et al.* 1993, Koopman 2002). Raw CRC32, however, is not designed for collision‐free hashing of genomic k-mers: although the theoretical capacity matches the number of possible 16-mers, collisions remain common for *k ≥ *5 in real datasets due to non‐ideal polynomial choices and the absence of input‐specific preprocessing (Ondov *et al.* 2016, Wood *et al.* 2019).

Here, we introduce genCRC32, a collision‐free hashing scheme for DNA k-mers of length up to 16 bases. Our method employs a lightweight bit‐level preprocessing step (“gen32”) that encodes each nucleotide into four bits and packs adjacent bases into bytes, transforming the original k-mer into a ⌈*k*/2⌉-byte intermediate. By aligning the 416 possible 16-mers with the 232 CRC32 output space and empirically identifying CRC32 polynomials that yield zero collisions, genCRC32 achieves a one‐to‐one mapping without auxiliary lookup tables. Importantly, genCRC32 is non‐locality preserving—similar sequences do not necessarily produce similar hashes—which mitigates clustering of related k-mers and can improve discrimination in applications such as variant detection and metagenomic classification (Hosseini *et al.* 2019, Jafari *et al.* 2021).

The simplicity of genCRC32’s preprocessing and the ubiquity of hardware‐accelerated CRC32 implementations ensure that our approach can be seamlessly integrated into existing bioinformatics pipelines. In the following sections, we detail the gen32 preprocessing algorithm, the selection of collision‐free CRC32 polynomials, and performance benchmarks comparing genCRC32 to standard CRC32, MurmurHash3, ntHash, PHast, and xxHash32 on exhaustive k-mer sets. We evaluate genCRC32 as a hashing primitive on these k-mer sets and report collision behavior, bucket balance, sensitivity to single-base changes, and speed. Evaluation within specific software tools is planned for future work.

## 2 Methods

### 2.1 Algorithm implementation

We developed genCRC32 in Go (version 1.24) using only the standard library to ensure portability and simplicity. The pipeline comprises a bit-level preprocessing step (gen32) that packs nucleotide string into a byte-level representation, followed by a CRC32 checksum computed via Go’s hash/crc32 package. The code is compiled with the default go build command (no additional flags are necessary).

### 2.2 Preprocessing (gen32)

The gen32 function converts a DNA sequence of length *k* into a byte array of ⌈*k*/2⌉ bytes. No explicit case normalization is needed: masking each character with 0x0E inherently yields identical codes for uppercase and lowercase nucleotides by isolating bits 1–3 of the ASCII byte, which differ uniquely among A/a, C/c, G/g, and T/t. Two resulting 4-bit codes are concatenated into one byte. The pseudocode for the gen32 function is:

*function gen32_preprocessing(sequence):*

*k ← length(sequence)*

*size ← (k ÷ 2) + (k mod 2)*

*result ← make([]byte, size) # all bytes zero*

*for i from 0 to k − 1:*

*# Extract 4-bit nucleotide code via ASCII masking:*

*# ′A′/′a′ → 0x00, ′C′/′c′ → 0x02, ′G′/′g′ → 0x06, ′T′/′t′ → 0x04*

*code ← sequence[i] AND 0x0E*

*# Shift existing nibble to high 4 bits*

*result[i ÷ 2] ← result[i ÷ 2] ≪ 4*

*# Insert new code in low 4 bits*

*result[i ÷ 2] ← result[i ÷ 2] OR code*

*return result*

By packing two bases per byte, this step encodes all 4^k possible k-mers into ⌈*k*/2⌉ bytes—i.e. approximately half the original byte length (e.g. a 16-mer → 8 bytes = 64 bits).

### 2.3 CRC32 hashing and polynomial selection

We evaluated twenty 32-bit CRC polynomials—commonly used in hardware and software CRC32 implementations (Castagnoli *et al.* 1993, Koopman 2002, Koopman and Chakravarty 2004)—including: 0xEB31D82E, 0xEDB88320, 0x82F63B78, 0xAD0424F3, 0x945D045D, 0x9D9947FD, 0xA3000000, 0x87496166, 0x8E2371EF, 0x8C746ED4, 0x8EFD4BCD, 0x80000057, 0x8741C726, 0x82608EDB, 0x8EE5368F, 0xE47B4C57, 0xD2C0EF07, 0x80FCB077, 0x93B39B1B, 0xB338ADD6.

Through exhaustive testing, we identified eight polynomials—0x8741C726, 0x87496166, 0x8E2371EF, 0x8EE5368F, 0x8EFD4BCD, 0x945D045D, 0x9D9947FD, and 0xEB31D82E—that yield zero collisions for all gen32 preprocessed k-mers up to length 16.

### 2.4 Collision testing and benchmarking

For each *k* ∈ {4…16}, we generated the complete set of 4ᵏ k-mers and applied eight different hashing protocols:

Raw CRC32 (all 20 polynomials)

gen32 + CRC32 (same polynomials)

Raw MurmurHash3 (via github.com/spaolacci/murmur3)

gen32 + MurmurHash3

Raw xxHash32 (via https://github.com/OneOfOne/xxhash)

gen32 + xxHash32

ntHash v2.3.0 (https://github.com/bcgsc/ntHash)

PHast v0.10.0 (https://docs.rs/crate/ph)

Every hash value was inserted into an in-memory Go map (dictionary/set) to detect collisions. Each configuration (hash function × polynomial) was run in randomized order and repeated 10×times. Because collisions were identical across replicates, we report the mean collision count (which equals the exact count per run) for each configuration.

Throughput was computed as the total number of k-mers hashed divided by the elapsed wall time for the exhaustive collision-testing runs (for each *k*), and we report it as hashes per second (H/s or MH/s).

### 2.5 Uniformity across buckets

We evaluated how well each hashing method distributes outputs by measuring bucket uniformity on three input regimes representative of common k-mer workloads. Each regime comprised 1 000 000 k-mers: (i) Random, uniformly sampled k-mers; (ii) Similar, near-neighbor variants generated by single-base edit of previous k-mer; and (iii) Window, overlapping k-mers obtained with a sliding window from the *Escherichia coli* K-12 MG1655 reference (NC_000913.3). The identical k-mer lists were hashed by all methods to isolate hashing behavior from input composition.

For each method and dataset we mapped hash values to m buckets via bucket = h mod m, using a panel of bucket counts spanning typical hash-table sizes (powers of two and nearby odd primes). We tallied bucket occupancies and computed the Pearson χ^2^ statistic against the ideal count n/m, converting to *P* values (*df* = *m* − 1). To ensure the validity of χ^2^ approximations, we restricted values to bucketings with expected counts > 5.

### 2.6 Avalanche effect

We quantified output diffusion using the Random k-mer set (same as above). For each original k-mer we generated three independent single-nucleotide substitutions (one base changed per variant), yielding three origin–mutation pairs per k-mer. Each method hashed all originals and all mutated counterparts. For every pair, we computed the XOR of the two hash values and measured the fraction of flipped output bits (Hamming distance divided by the hash width *b*, with *b* = 32 for 32-bit methods). Our summary metric is mean flip, the average of this fraction across all pairs. An ideal avalanche property corresponds to mean flip = 0.5.

### 2.7 Performance measurement and environment

We measured elapsed real time for each full k-mer hashing run using Go’s time package. All benchmarks were executed on a single workstation (Intel Xeon E5-1630 v3 CPU, 64 GB RAM, Crucial MX500 500 GB SSD, Ubuntu 20.04 LTS).

## 3 Results

### 3.1 Collision-free performance of genCRC32

The genCRC32 algorithm combines a novel preprocessing method (gen32) with the CRC32 hashing algorithm. We systematically tested its collision-free capability across a range of k-mer lengths (*k = *4–16). Using gen32 preprocessing, eight CRC32 polynomials (0x8741C726, 0x87496166, 0x8E2371EF, 0x8EE5368F, 0x8EFD4BCD, 0x945D045D, 0x9D9947FD, and 0xEB31D82E) demonstrated complete collision-free performance for the entire tested range (*k = *4–16). ntHash and PHast were included as comparators. Being collision-free by design, they did not require gen32 preprocessing in our evaluations. By contrast, all other tested hash configurations, including raw CRC32, MurmurHash3 and xxHash32 with and without gen32 preprocessing, exhibited collisions for one or more of the k-mer lengths evaluated (Table 1).

**Table 1.** Collisions observed for different hash configurations and *k*.

Hash configuration	Collision-free k value range	*k* value range with collisions
gen32 + MurmurHash3 Raw MurmurHash3	4–8	9–16
Raw xxHash Raw CRC32 (D2C0EF07)	4–9	10–16
gen32 + xxHash Raw CRC32 (80FCB077, 8741C726, 8C746ED4, 8EE5368F, 945D045D)	4–10	11–16
Gen32 + CRC32 (82f63b78) Raw CRC32 (80000057, 82608EDB, 82F63B78, 87496166, 8E2371EF, 8EFD4BCD, 93B39B1B, 9D9947FD, A3000000, AD0424F3, B338ADD6, E47B4C57, EB31D82E, EDB88320)	4–11	12–16
gen32 + CRC32 (93B39B1B)	4–13	14–16
gen32 + CRC32 (B338ADD6)	4–14	15–16
gen32 + CRC32 (80000057, 80FCB077, 82608EDB, 8C746ED4, A3000000, AD0424F3, D2C0EF07, E47B4C57, EDB88320)	4–15	16
gen32 + CRC32 (8741C726, 87496166, 8E2371EF, 8EE5368F, 8EFD4BCD, 945D045D, 9D9947FD, EB31D82E) ntHash PHast	4–16	–

It is notable that *k = *16 represents the maximum theoretical capacity of a 32-bit hash space, thus making collision-free performance at this length particularly significant.

### 3.2 Execution performance and speed

Execution speed benchmarks revealed that the gen32 preprocessing step introduces only minimal computational overhead compared to direct hashing without preprocessing. Specifically, the average hashing speed across all tested configurations (*k = *4–16) was 23 245 622 hashes per second with preprocessing, compared to 25 031 005 hashes per second without preprocessing (Supplementary Table 1). This indicates that gen32 preprocessing imposes negligible overhead, maintaining efficient hashing even at the largest tested k-mer length (*k = *16).

Detailed analysis of execution speed at *k = *16 showed mean speeds of 31 124 560 hashes per second (with gen32) and 31 622 151 hashes per second (without preprocessing). This negligible difference in speed is crucial since *k = *16 is the critical limit for collision-free 32-bit hashing, underscoring the efficiency of gen32 preprocessing at maximum capacity. Table 2 reports throughput at *k = *16, computed as the total number of k-mers hashed divided by the elapsed wall time for the exhaustive runs. Across CRC32 variants, MurmurHash3, and xxHash32, observed throughputs were of the same order of magnitude, and adding gen32 preprocessing incurred only marginal overhead relative to the corresponding raw variant. In our measurements, ntHash attained the highest throughput, consistent with its rolling formulation optimized for adjacent k-mers, whereas PHast was the slowest among the tested implementations. We caution that absolute speed is implementation-sensitive (language, vectorization, memory layout, engineering choices), so the figures should be read as descriptive under our setup; the key observation is that gen32 does not materially affect throughput within our pipeline.

**Table 2.** Hashing metrics and throughput at *k = *16: bucket-occupancy uniformity (χ^2^  *P* values for Random/Similar/Window), avalanche effect (mean flip; ideal = 0.5), and throughput (M hash/s).

		Bucket-occupancy uniformity				Avalanche effect	
Algorithm	Preprocessing	*P* value (random)	*P* value (similar)	*P* value (window)	Mean flip		Speed MH/s
CRC32 (8741C726)	gen32	.53	.86	.13	0.48		33.24
CRC32 (8741C726)	None	.41	.51	.13	0.50		30.51
CRC32 (87496166)	gen32	.32	.87	.14	0.51		33.28
CRC32 (87496166)	None	.41	.60	.20	0.51		30.24
CRC32 (8E2371EF)	gen32	.46	.80	.16	0.51		33.31
CRC32 (8E2371EF)	None	.59	.70	.06	0.51		30.60
CRC32 (8EE5368F)	gen32	.50	.88	.14	0.51		33.35
CRC32 (8EE5368F)	None	.32	.65	.12	0.50		30.50
CRC32 (8EFD4BCD)	gen32	.55	.80	.09	0.45		33.27
CRC32 (8EFD4BCD)	None	.46	.67	.25	0.49		30.45
CRC32 (945D045D)	gen32	.65	.96	.11	0.48		33.25
CRC32 (945D045D)	None	.43	.57	.20	0.48		30.39
CRC32 (9D9947FD)	gen32	.40	.83	.22	0.50		33.30
CRC32 (9D9947FD)	None	.40	.73	.22	0.51		30.72
CRC32 (EB31D82E)	gen32	.42	.86	.11	0.49		33.70
CRC32 (EB31D82E)	None	.46	.70	.10	0.50		30.45
MurmurHash3	gen32	.43	.57	.04	0.50		29.84
MurmurHash3	None	.37	.45	.11	0.50		35.73
xxHash	gen32	.33	.25	.09	0.50		29.75
xxHash	None	.50	.52	.13	0.50		36.67
ntHash	None	.45	.42	.07	0.49		91.51
PHast	None	1.00	1.00	1.00	0.37		1.14

### 3.3 Scaling with k-mer length

**Figure 1.**
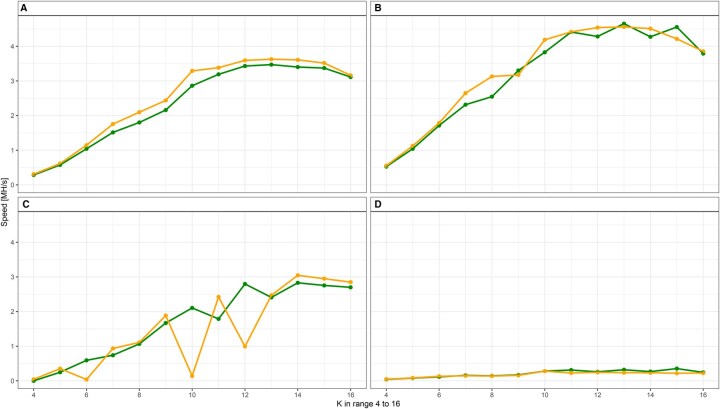
Four‐panel comparison of hashing performance across *k* values (4–16) on *X*-axis for configurations with gen32 preprocessing (orange line with markers) versus no preprocessing (green line with markers). (A) Average speed, (B) maximal speed, (C) minimal speed, and (D) standard deviation of speed.

Analyzing execution speed across different k-mer lengths (*k = *4–16; Fig. 1), we observed consistent linear scaling of hashing speed with increasing k-mer length. The observed lower speeds at smaller k-mer lengths (*k* ≤ 10) are likely due to general computational overhead and external system processes influencing short-duration tasks. Both preprocessed and raw hashing configurations followed similar trends, with preprocessing consistently showing slightly lower yet comparable speeds. Differences in execution speeds diminished substantially as k-mer length increased (*k ≥ *10), confirming minimal preprocessing overhead for larger datasets.

### 3.4 Consistency and robustness

Throughout our experiments, collision counts remained constant across multiple replicates, demonstrating the robust and deterministic behavior of all tested hashing configurations. This consistency confirms the stability of genCRC32's performance, rendering it highly suitable for reliable integration into bioinformatics pipelines.

### 3.5 Bucket-occupancy uniformity and avalanche effect

Table 2 summarizes three properties: (i) speed described above (ii) bucket-occupancy uniformity, reported as dataset-level *P* values for the Random, Similar, and Window inputs (Pearson’s χ^2^ goodness-of-fit against the uniform expectation), and (iii) the avalanche effect, reported as mean flip. These metrics reflect, respectively, how evenly hash values populate buckets after modulo reduction and how sensitively (and uniformly) output bits respond to small input perturbations.

In our measurements, gen32 + CRC32 tended to yield higher uniformity *P* values than the corresponding raw CRC32 configurations on the Similar dataset—consistent with more balanced bucket occupancies under localized edits—while behavior on Random was broadly comparable and Window appeared most challenging across methods. For avalanche effect, most general-purpose hashes (CRC32 variants, MurmurHash3, xxHash32, ntHash) exhibited mean flip values close to 0.5. By contrast, PHast showed lower mean flip despite very even bucket counts, which aligns with the design goals of minimal/perfect hashing—injective placement of a fixed key set into a fixed address range (e.g. bucket-then-seed schemes)—prioritizing collision-free, highly regular placement rather than per-bit diffusion.

### 3.6 Practical utility in hash-based pipelines

Many k-mer–centric pipelines (e.g. exact-match indices, hash-table-based k-mer stores, and sketching/seeding components) require more than a simple symbol-to-bit transformation and may benefit from bucketization, diffusion under small edits, and predictable throughput. In this setting, genCRC32 provides a deterministic, collision-free mapping for all 4^k inputs with *k* ≤ 16 under our CRC32 configurations, exhibits balanced bucket occupancies across representative bucketings, shows near-ideal diffusion under single-base edits (mean flip ≈ 0.5), and attains throughput comparable to standard CPU-friendly hashes with only marginal preprocessing overhead. These properties address the practical factors that govern load balance, robustness, and runtime cost in hash-based genomics tooling, complementing compact 2-bit encodings (storage-oriented) and key-set–specific perfect hashing (placement-oriented) by characterized hashing behavior directly.

### 3.7 Summary of importance

The collision-free performance, combined with minimal computational overhead, confirms the practical utility of genCRC32 in bioinformatics. Its robustness, consistent speed, and capability to achieve theoretical maximum collision-free performance at *k = *16 make it highly advantageous for efficient and precise management of genomic datasets in various computational genomics applications.

## 4 Discussion

In this study, we introduced genCRC32, an innovative hashing approach tailored specifically for bioinformatics applications that require precise, collision-free hashing of DNA sequences. By integrating a straightforward preprocessing step (gen32) with carefully selected CRC32 polynomials, we successfully achieved collision-free hashing performance for all possible k-mers up to the theoretical maximum length of *k = *16 for a 32-bit hash space.

Our identification of eight CRC32 polynomials that provide collision-free hashing was strictly empirical. Currently, there is no established theoretical explanation for why these specific polynomials perform this way, especially considering the preprocessing step transforms nucleotide sequences into intermediate binary representations. It is probable that other (non-tested) CRC32 polynomials could also provide collision-free hashing performance. However, there is currently no basis to believe they would offer any advantage over the eight polynomials identified in this study. Future research could benefit from mathematical or computational studies designed explicitly to explore polynomial performance in hashing tasks.

Collision-free hashing performance at *k* ≤ 16 is particularly valuable in genome assembly and alignment applications, where collision-induced ambiguities can significantly impair accuracy or performance of downstream analyses (Phillippy *et al.* 2008, Bankevich *et al.* 2012). Because assemblers and k-mer classifiers use the hash as an internal primitive, the key determinants are the hash’s collision rate, bucket balance, diffusion to single-base edits, and throughput; we therefore evaluate on exhaustive k-mer sets and representative workloads. For instance, de Bruijn graph-based genome assemblers such as SPAdes and Velvet rely on precise k-mer identification, directly affecting graph complexity and assembly accuracy (Zerbino and Birney 2008, Bankevich *et al.* 2012). Similarly, taxonomic classifiers like Kraken2 depend heavily on precise hashing to accurately assign metagenomic sequences, especially when handling complex and extensive reference databases (Wood *et al.* 2019, Lu and Salzberg 2020). In comparison, commonly used hash functions like MurmurHash3 and xxHash32, while computationally efficient, do not guarantee collision-free hashing, highlighting the practical advantage of genCRC32 in scenarios demanding high accuracy and speed.

Hashing is also foundational to minimizer-based sampling of k-mers, where a (pseudo)random hash induces an order within each window and the minimal element is retained as the seed. Minimizers and recent variants (e.g. mod-minimizers) are widely used in seeding, indexing, and sketching (Koerkamp and Pibiri 2024). An interesting direction for future work is to examine how the distributional properties of candidate hash functions (e.g. near-uniformity and independence across k-mers) influence the induced order and sampling density in minimizer schemes.

Nevertheless, it is crucial to recognize genCRC32’s limitations when considering k-mer lengths beyond *k = *16. Given the empirical nature of our polynomial discovery, extending collision-free hashing to larger k-mers would require larger hash spaces and/or alternative hashing methodologies. However, for typical bioinformatics applications, including genome assembly, variant detection, and rapid indexing in metagenomics, collision-free hashing at *k* ≤ 16 is sufficiently robust and practical (Ondov *et al.* 2016, Marçais *et al.* 2018, Lu and Salzberg 2020). For *k* ≤ 16, the four-letter alphabet admits an injective identifier via direct 2-bit encoding (a 2k-bit state). This representation would support O(1) rolling updates (left-shift by two bits, retain the last 2k bits, then append the incoming base) and thus offers a collision-free alternative orthogonal to our CRC32 design. We did not implement this variant here; we note it as a feasible option for applications that specifically require rolling updates or guaranteed injectivity at small *k*.

A significant strength of genCRC32 lies in its simplicity and ease of integration. Its straightforward preprocessing step, combined with widely supported and optimized CRC32 hashing implementations, facilitates seamless integration into existing bioinformatics workflows, minimizing disruption and overhead. This practical advantage is particularly beneficial for tools and pipelines already operational in research and production environments.

In conclusion, genCRC32 represents a practical and effective advancement in bioinformatics hashing methodologies, achieving collision-free hashing performance at the theoretical maximum limit for 32-bit hashing. While further theoretical exploration into polynomial selection would be valuable, the current implementation already delivers substantial practical benefits. genCRC32 provides fast, robust, reliable, and collision-free hashing performance suitable for precise and high-throughput bioinformatics applications, highlighting its immediate and practical applicability in modern computational genomics.

## Supplementary Material

vbaf315_Supplementary_Data

## Data Availability

Source code, documentation and executable binary files are available at the GitHub repository (https://github.com/berybox/genCRC32).
