# Supplementary material for: genCRC32: collision-free CRC32-based hashing of DNA sequences
Source: Bioinform Adv. 2025 Dec 4;6(1):vbaf315. doi: 10.1093/bioadv/vbaf315 (PMC12996896; doi:10.1093/bioadv/vbaf315)
Supplement: vbaf315_Supplementary_Data [file vbaf315_supplementary_data.docx]

**Supplementary Table 1.** Summarized hashing speed by k values for hash configurations with and without gen32 preprocessing. Showing mean number of hashes per second for each hashing configuration + K

| **k** | **Preprocessor** | **Mean speed (Hash / s)** |
| --- | --- | --- |
| 4 | gen32 | 2858199.34 |
| 4 | None | 3097158.61 |
| 5 | gen32 | 5767516.66 |
| 5 | None | 6154619.83 |
| 6 | gen32 | 10410612.21 |
| 6 | None | 11461766.86 |
| 7 | gen32 | 15147499.71 |
| 7 | None | 17553266.81 |
| 8 | gen32 | 18017268.22 |
| 8 | None | 20984238.17 |
| 9 | gen32 | 21601637.92 |
| 9 | None | 24376918.04 |
| 10 | gen32 | 28617408.95 |
| 10 | None | 32894766.77 |
| 11 | gen32 | 31907949.29 |
| 11 | None | 33819536.10 |
| 12 | gen32 | 34305276.05 |
| 12 | None | 35930664.08 |
| 13 | gen32 | 34704956.69 |
| 13 | None | 36267270.97 |
| 14 | gen32 | 34013763.97 |
| 14 | None | 36079607.08 |
| 15 | gen32 | 33716435.36 |
| 15 | None | 35161094.36 |
| 16 | gen32 | 31124560.17 |
| 16 | None | 31622151.45 |
